# Federated Learning and Data Mining-Based Botnet Attack Detection Framework for Internet of Things

**DOI:** 10.3390/s26051573

**Published:** 2026-03-02

**Authors:** Kalupahana Liyanage Kushan Sudheera, Lokuge Lehele Gedara Madhuwantha Priyashan, Oruthota Arachchige Sanduni Pavithra, Malwaththe Widanalage Tharindu Aththanayake, Piyumi Bhagya Sudasinghe, Wijethunga Gamage Chatum Aloj Sankalpa, Gammana Guruge Nadeesha Sandamali, Peter Han Joo Chong

**Affiliations:** 1 Department of Electrical and Information Engineering, Faculty of Engineering, University of Ruhuna, Galle 80000, Sri Lanka; eg163032@engug.ruh.ac.lk (L.L.G.M.P.); eg162819@engug.ruh.ac.lk (O.A.S.P.); eg162822@engug.ruh.ac.lk (M.W.T.A.); piyumi.s@eie.ruh.ac.lk (P.B.S.); chatum@eie.ruh.ac.lk (W.G.C.A.S.); nadeesha@eie.ruh.ac.lk (G.G.N.S.); 2 School of Engineering, Computer and Mathematical Sciences, Auckland University of Technology, Auckland 1010, New Zealand; peter.chong@aut.ac.nz

**Keywords:** botnet attack, cyber-security, data mining, federated learning, internet of things, machine learning

## Abstract

Botnet attacks in Internet of Things (IoT) environments often occur as multi-stage campaigns, making early and reliable detection difficult across distributed and privacy-sensitive networks. Centralized detection approaches are often limited by heterogeneous traffic characteristics, severe data imbalance, and the need to aggregate large volumes of raw network data, raising scalability and privacy concerns. To address these challenges, this paper proposes FDA, a federated learning-based and data mining-driven framework for stage-aware botnet attack detection in IoT networks. FDA operates at network gateways, where anomalous traffic is first detected and then abstracted into compact and interpretable patterns using Frequent Itemset Mining (FIM). This pattern-based representation reduces noise and local traffic bias, enabling more robust learning across different IoT networks. Lightweight neural network models are trained locally at gateways, and a global model is learned through federated aggregation of model parameters, avoiding direct sharing of raw network data while enabling gateways to collaboratively learn evolving attack patterns across different IoT networks. Experimental results show that FDA achieves anomaly detection F1-scores above 99% across all gateways and multi-stage botnet attack classification F1-scores in the range of 48–49%, which are comparable to centralized machine-learning baselines while operating under decentralized and privacy-preserving constraints. Overall, FDA provides a practical, privacy-preserving, and effective solution for distributed botnet attack stage detection in real-world IoT deployments.

## 1. Introduction

The Internet of Things (IoT) has experienced rapid growth over the past decade and is now widely deployed across residential, industrial, and smart-city environments. According to [1], the global IoT market is projected to approach USD 2500 billion by 2029. IoT systems enable remote access and real-time communication among interconnected devices, offering significant convenience and automation. Common examples include Closed-Circuit Television (CCTV) cameras, which allow users to monitor live video streams remotely. However, alongside these benefits, the widespread adoption of IoT technologies has led to the deployment of highly heterogeneous and often non-standard devices and endpoints [2].

Many IoT devices are deployed with limited security mechanisms and are rarely patched after installation. As a result, they frequently expose critical services through open ports or default credentials, making them vulnerable to cyber-attacks. Given their sheer number and diversity, IoT devices present an attractive and scalable attack surface for adversaries. This has been clearly demonstrated by large-scale botnet attacks such as Mirai and Hajime [3]. In particular, the Mirai botnet compromised more than 600,000 vulnerable IoT devices and launched high-impact distributed denial-of-service (DDoS) attacks against prominent targets, including Krebs on Security and Dyn, a major DNS service provider for platforms such as Amazon, GitHub, Netflix, PayPal, Reddit, and Twitter [3].

Botnet attacks in IoT environments typically consist of multiple attack stages that unfold across different networks and over extended periods of time, making them both spatially and temporally dispersed. A typical botnet campaign begins with reconnaissance, during which attackers scan IoT devices for vulnerabilities. Once vulnerable devices are identified, subsequent stages such as intrusion, malware injection, command-and-control (C&C) communication, and targeted attacks—including SYN flooding, DDoS, and data exfiltration—are executed. Although these stages generate observable traces in network traffic, manually identifying and correlating such traces over time is impractical, particularly in large-scale IoT deployments.

To address these challenges, effective detection mechanisms are required to capture and correlate different botnet attack stages in a timely manner. Such mechanisms can provide early warnings of malware propagation, enable attack-stage prediction, and support rapid mitigation [4]. Endpoint-based detection solutions are often unsuitable for IoT environments, as many devices are resource-constrained and are not regularly updated [5]. In contrast, network-level detection approaches offer broader visibility and can learn from traffic patterns across multiple devices and attack variants. However, centralized network-level solutions typically require aggregating user-generated traffic data, raising significant privacy concerns. Prior studies have shown that even encrypted traffic can reveal sensitive behavioral information about users [5,6].

In this work, a FDA is proposed for identifying individual stages of botnet attacks in IoT networks. FDA is a network-level solution deployed across local gateways and a centralized network manager. At each gateway, network traffic is first filtered using an anomaly detection mechanism to identify anomalous flows. These anomalies are then transformed into compact and interpretable representations using frequent itemset mining (FIM), followed by association rule extraction that links network features to specific botnet attack stages. The resulting association rules are used to train lightweight neural network models locally at gateways.

To enable collaborative learning across distributed IoT networks without directly sharing raw traffic data, FDA employs an FL strategy in which only model parameters are exchanged with the centralized network manager. This allows the global model to learn from diverse attack patterns observed across multiple networks while limiting direct exposure of user data. The proposed framework is evaluated using an OpenStack-based emulation environment, where results demonstrate that FDA achieves detection performance comparable to centralized approaches. In addition, a real IoT testbed is used to validate the practical applicability of the framework under realistic conditions.

The main contributions of this paper are summarized as follows:

- We propose FDA, a novel stage-aware and privacy-preserving botnet attack detection framework for IoT networks that integrates anomaly detection, frequent itemset mining, association rule analysis, and federated learning.
- We introduce a pattern-level abstraction mechanism that transforms raw anomalous traffic into compact and interpretable representations, reducing noise and local traffic bias.
- We design a lightweight neural network model trained locally at gateways and aggregated using federated learning to enable collaborative multi-stage attack classification without sharing raw traffic data.
- We evaluate the proposed framework using both an OpenStack-based emulation environment and a real IoT testbed to demonstrate its effectiveness and practicality.

The remainder of this paper is organized as follows. Section 2 reviews related work on IoT intrusion detection and multi-stage attack analysis. Section 3 presents the background and threat model. Section 4 describes the FDA framework in detail. Section 5 discusses the experimental setup and evaluation results, and Section 6 concludes the paper.

## 2. Related Work

Research on securing IoT networks against botnet attacks spans intrusion detection, attack-stage analysis, and distributed learning paradigms. This section reviews existing work in these areas and highlights the limitations that motivate the design of FDA. For each closely related study, we briefly highlight the main technique and its key limitation with respect to stage-aware and privacy-preserving botnet detection.

### 2.1. Intrusion Detection in IoT Networks

A large body of work has explored machine-learning-based intrusion detection systems (IDSs) for IoT environments. Anthi et al. [7] proposed a supervised IDS architecture comprising three layers: device fingerprinting, anomaly detection, and attack classification. Although this approach demonstrates strong detection performance, it relies on centralized training and packet-level features collected from a single network, which limits scalability and generalization across heterogeneous IoT deployments. Moreover, this approach does not explicitly support detection of individual botnet attack stages.

Unsupervised and semi-supervised methods have been proposed to reduce dependency on labeled data. Nomm and Bahsi [8] introduced an anomaly-based botnet detection approach that selects features using entropy and variance measures to reduce computational overhead. While effective in identifying unknown attacks, such methods are prone to high false-positive rates, as benign behavioral deviations are often misclassified as malicious. This limits their practicality in real-world IoT environments, where traffic patterns are highly dynamic. In addition, these methods focus mainly on binary anomaly detection rather than stage-level classification.

To improve responsiveness, fog- and edge-based detection approaches have also been investigated. Samy et al. [9] proposed a fog-assisted framework in which traffic is captured at gateways and forwarded to the cloud for training a Long Short-Term Memory (LSTM) model, which is then deployed at the fog layer for inference. Despite reduced detection latency, this approach requires centralized training on raw traffic data, incurs high computational overhead, and raises privacy concerns due to cross-domain data sharing. This motivates the need for decentralized and privacy-aware learning mechanisms.

### 2.2. Detection of Individual Botnet Attack Stages

Several studies focus on detecting specific stages of botnet attacks rather than the complete attack lifecycle. Early-stage reconnaissance has been addressed using deep learning techniques to identify scanning behavior and abnormal connection patterns [10]. Intrusion attempts and unauthorized access have been studied using login behavior analysis and NetFlow-based features, particularly for detecting SSH-based attacks [11,12]. However, these works typically consider stages in isolation and do not provide an integrated multi-stage detection framework.

Command-and-control (C&C) communication, a critical component of botnet operations, has been investigated in systems such as BotDet [13], which detects C&C traffic using indicators including blacklisted IP addresses, suspicious domains, SSL certificates, and TOR usage. Alerts generated from multiple indicators are correlated to reduce false positives. While effective, these methods are tailored to specific C&C behaviors and require frequent updates to maintain accuracy. They also rely on centralized analysis of traffic features.

DDoS detection in IoT networks has also received significant attention. In [14], inherent IoT characteristics such as limited communication endpoints and periodic traffic patterns were leveraged to train an ML-based DDoS detector. Similarly, volumetric attack detection using Software Defined Networking (SDN) and Manufacture Usage Description (MUD) profiles was explored in [15]. Although these approaches achieve strong performance for specific attack types, they operate in isolation and do not correlate evidence across multiple attack stages. Consequently, early-stage attack visibility remains limited.

### 2.3. Multi-Stage Attack Detection and Correlation

Detecting coordinated, multi-stage attacks requires correlating alerts and traffic patterns over time. Alert correlation techniques based on probabilistic similarity estimation and sequential pattern mining have been proposed in [16,17]. Hidden Markov Models (HMMs) were employed in [18] to infer attack stages by modeling alerts as observable states. However, such approaches rely on predefined state transitions, which may not generalize well to evolving botnet behaviors and adaptive attack strategies. In addition, these techniques are commonly deployed in centralized settings.

Clustering-based approaches have also been explored to identify multi-stage attacks by grouping related traffic flows [19]. While these methods can uncover latent attack structures, they do not explicitly map discovered clusters to semantic attack stages, limiting their operational interpretability.

Our prior work, ADEPT [20], proposed a distributed framework for detecting and identifying individual stages of coordinated botnet attacks in IoT networks. ADEPT performs anomaly detection at gateways and forwards alerts to a centralized security manager, where spatial and temporal correlations are mined to train an ML-based classifier. Although ADEPT avoids direct exposure of raw packet payloads, packet-level metadata are still shared, and recent studies have shown that such metadata can be exploited to infer sensitive user behavior [21]. This motivates further reduction of shared information.

### 2.4. Privacy-Aware and Distributed Learning Approaches

Federated learning has emerged as a promising paradigm for collaborative model training without centralized data aggregation. Early formulations of federated optimization demonstrated its feasibility for on-device learning under privacy constraints [22]. More recent studies have explored its application to network security and anomaly detection. However, most existing works apply federated learning directly to raw or minimally processed features, which remain sensitive and susceptible to inference attacks.

FDA differs from existing approaches by combining pattern-level abstraction with federated learning. Instead of sharing raw traffic features or alerts, FDA performs frequent itemset mining and association rule extraction locally at gateways, producing compact and interpretable representations that reduce noise and local traffic bias. Model training is also performed locally, and only model parameters are exchanged during federated aggregation. This design enables stage-aware botnet detection across distributed IoT networks while mitigating privacy risks associated with centralized or metadata-sharing approaches.

Overall, compared with existing approaches, FDA uniquely combines stage-aware botnet detection, pattern-level abstraction, and federated learning to achieve privacy-preserving multi-stage attack analysis across distributed IoT networks.

## 3. Background

### 3.1. Threat Model

The rapid proliferation of IoT deployments has significantly expanded the attack surface available to adversaries, leading to a corresponding rise in botnet-based cyber-attacks. In this context, a bot refers to a compromised IoT device that is remotely controlled as part of a botnet infrastructure. A botnet typically consists of three core components: a collection of infected devices (bots), one or more command-and-control (C&C) servers, and a botmaster who orchestrates malicious activities.

Many IoT devices are deployed with minimal security hardening and remain unpatched throughout their operational lifetime. Common vulnerabilities include exposed services such as SSH and Telnet configured with default credentials, which make these devices attractive targets for automated compromise [23]. Once infected, compromised devices actively propagate malware by scanning and exploiting other vulnerable IoT devices, thereby expanding the botnet. Control over infected devices is maintained through persistent C&C communication channels.

Recent IoT botnets such as Mirai and Hajime exemplify the scale and impact of these threats. Mirai demonstrated the feasibility of launching large-scale DDoS attacks by compromising IP cameras, DVRs, and routers, culminating in an attack exceeding 1 Tbps against the Dyn DNS infrastructure [3]. Hajime employs a similar infection strategy but differs in its decentralized architecture, leveraging BitTorrent Distributed Hash Tables (DHTs) and peer-to-peer communication to eliminate centralized C&C points [24].

Botnet campaigns typically unfold as multi-stage processes that are distributed across networks and time [25].

**Figure 1:**
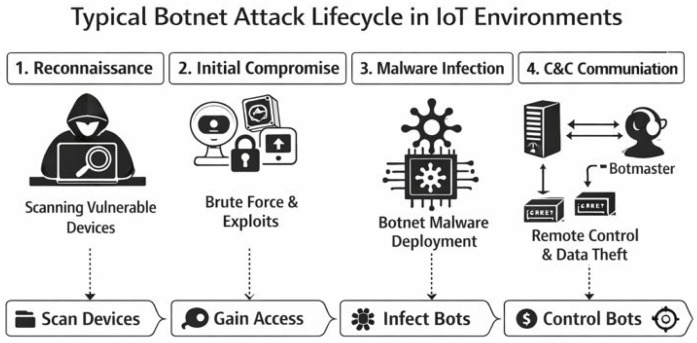
Typical botnet attack lifecycle in IoT environments.

Figure 1 illustrates the typical botnet attack lifecycle in IoT environments, highlighting the progression from reconnaissance and login to malware infection, command-and-control (C&C) communication.

Accordingly, although specific implementations vary across botnet families, the attack lifecycle can be generalized into the following stages:

- **Reconnaissance:** Bots scan the Internet to identify vulnerable IoT devices, primarily by probing for exposed services such as SSH and Telnet.
- **Login:** Identified devices are targeted using dictionary-based brute-force authentication attempts.
- **Malware Infection:** Upon successful access, malware binaries are downloaded from external servers and installed to ensure persistence.
- **C&C Communication:** Infected devices establish outbound communication with C&C infrastructure to receive commands and transmit status updates, often using periodic heartbeat messages.
- **Targeted Attacks:** Coordinated attacks, most commonly DDoS, are launched against designated victims using traffic generated from compromised IoT devices. These attacks may include volumetric floods, protocol-level exploits, or application-layer attacks [26].

For example, in a Mirai-like botnet campaign, infected devices initially perform large-scale scanning to discover IoT nodes exposing services such as Telnet or SSH. Once a vulnerable device is found, dictionary-based brute-force login attempts are launched using default or weak credentials. After successful authentication, a malware binary is downloaded from an external server and executed, allowing the device to establish persistent C&C communication. The compromised device then becomes part of the botnet and may participate in coordinated attacks such as distributed denial-of-service (DDoS) against designated victims.

FDA is designed to detect and distinguish these stages by identifying characteristic traffic patterns associated with each phase, rather than relying on single-event alerts.

### 3.2. Profiling IoT Devices

IoT devices typically exhibit stable and predictable communication behavior, interacting with a limited set of endpoints using a narrow range of protocols defined by manufacturers. These characteristics enable the construction of benign behavioral profiles that serve as references for anomaly detection. Device profiles are established during initial deployment or firmware updates and are assumed to reflect legitimate operation.

In FDA, benign profiles are stored locally at gateways and continuously consulted during runtime to identify deviations indicative of compromise. Given the access pattern dominated by frequent lookups and infrequent insertions, cuckoo hash tables are employed to store device profiles, following the design in [20]. This choice ensures constant-time lookup performance while maintaining acceptable insertion overhead.

### 3.3. Network Anomalies

Network anomalies are defined as deviations from expected communication behavior and may arise due to benign faults or malicious activity [27]. In IoT environments, anomalies caused by intrusions are of primary concern and form the basis of FDA’s initial filtering stage.

Two broad categories of anomalies are considered.

**Connection Anomalies**: Connection anomalies occur when an IoT device communicates with previously unseen or unauthorized endpoints. C&C communication during botnet operation is a representative example, as infected devices establish outbound connections to external servers that are not part of their normal communication profile.

**Behavioral Anomalies**: Behavioral anomalies arise when communication occurs over legitimate endpoints but deviates statistically from normal behavior. Examples include sudden increases in traffic volume or packet rates during DDoS participation. Although endpoints may be valid, the observed traffic patterns differ significantly from benign operation.

FDA uses detected anomalies as inputs for downstream pattern mining, enabling the abstraction of low-level deviations into higher-level representations.

### 3.4. Frequent Itemset Mining (FIM)

To transform raw anomalies into compact and interpretable representations, FDA employs Frequent Itemset Mining (FIM). FIM identifies recurring combinations of discretized network features that appear consistently across anomalous traffic instances. An itemset that satisfies a minimum support threshold is considered frequent and reflects a characteristic pattern of behavior.

Association rules derived from frequent itemsets encode conditional relationships between feature combinations and attack stages. These rules provide structured inputs for local model training and reduce sensitivity to noise and local traffic bias [28].

FDA adopts the Apriori algorithm for FIM due to its ability to explicitly measure support and confidence relationships between feature subsets [29]. Apriori iteratively constructs higher-order itemsets using join and prune operations, exploiting the property that infrequent itemsets cannot generate frequent supersets. This approach enables efficient mining of stage-relevant patterns from anomaly-filtered traffic.

### 3.5. Federated Learning

Conventional ML-based detection approaches rely on centralized data aggregation, which raises privacy concerns and conflicts with emerging data protection regulations [30]. Federated learning addresses this limitation by enabling collaborative model training without direct data sharing [22].

In FDA, lightweight neural network models are trained locally at gateways using pattern-level representations derived from FIM. Only model parameters are transmitted to a centralized network manager, where they are aggregated to form a global model. This design allows FDA to learn from diverse attack manifestations across distributed IoT networks while avoiding exposure of raw traffic data.

Although federated aggregation may not fully match the performance of centralized training, it provides a practical trade-off between detection accuracy and privacy preservation, aligning with the deployment constraints of real-world IoT environments.

Federated learning systems may be vulnerable to adversarial behaviors such as model poisoning, where malicious clients attempt to manipulate local updates to degrade global model performance. This work focuses on demonstrating the feasibility of combining federated learning with pattern-level representations for privacy-preserving botnet detection. Addressing robustness against adversarial participants and securing the aggregation process are important directions for future research. Potential extensions include robust aggregation strategies, client reputation mechanisms, and anomaly-based filtering of suspicious model updates.

### 3.6. System Assumptions

FDA is designed under the following practical assumptions. IoT devices are deployed behind network gateways capable of monitoring traffic metadata (e.g., flow-level features) but not packet payloads. Gateways are assumed to be trusted entities with sufficient computational resources to perform lightweight anomaly detection, pattern mining, and local model training. IoT devices themselves are not modified and do not participate directly in the learning process.

It is assumed that benign device communication profiles can be established during initial deployment or firmware updates, and that significant deviations from these profiles are indicative of abnormal behavior. Gateways are assumed to operate independently, observing traffic generated within their local administrative domains. A centralized network manager is available to coordinate federated aggregation, but it does not have access to raw traffic data or anomaly records. Communication between gateways and the network manager is assumed to be secure.

These assumptions reflect realistic IoT deployments and ensure that FDA can be applied without requiring changes to existing devices or violating data privacy constraints.

## 4. FDA-Proposed Framework

### 4.1. Problem Statement

The rapid expansion of IoT ecosystems has resulted in the widespread deployment of heterogeneous, third-party devices that often lack adequate security hardening. Many of these devices expose critical services such as SSH and Telnet with default credentials and receive limited post-deployment maintenance. As discussed in Section 3.1, such weaknesses are systematically exploited by botnets to execute multi-stage attacks in IoT environments.

The objective of this work is to detect and classify individual stages of botnet attacks at the network level while preserving user privacy. The proposed framework focuses on the attack stages defined in the threat model, including reconnaissance, login, malware infection, C&C communication, and targeted attacks. Although the discussion is limited to these stages, the framework is general and can be extended to other attack types given sufficient data.

### 4.2. Overview of FDA

**Figure 2:**
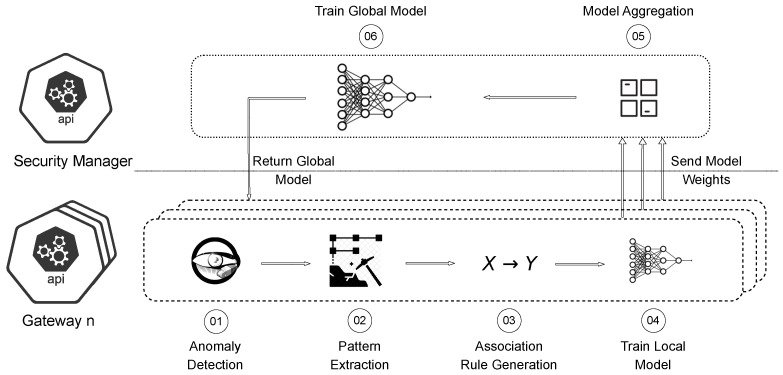
Architecture and operation of FDA.

FDA is a federated learning and data mining-based framework that combines pattern-level abstraction with collaborative model training to enable privacy-aware, stage-wise botnet attack detection. The overall architecture of FDA is illustrated in Figure 2.

A deployment scenario is considered comprising multiple distributed IoT networks, such as smart homes, each connected to a local gateway and overseen by a centralized security manager. Gateways monitor all ingress and egress traffic within their respective networks and are responsible for anomaly detection, pattern mining, and local model training. These gateways are assumed to be integrated with ISP-provided routers and equipped with sufficient computational resources comparable to modern mini-PCs [31].

The centralized security manager aggregates locally trained models to construct a global detection model. It can be deployed within an ISP data center or cloud infrastructure and integrated with security management platforms such as SIEM systems [32] or SDN controllers. The global model is periodically redistributed to gateways, enabling local attack-stage detection without continuous involvement of the centralized entity.

### 4.3. Anomaly Detection

As described in Section 3.3, FDA focuses on detecting *connection anomalies* and *behavioral anomalies*. Gateways passively monitor network traffic traversing the local IoT network and compare observed connections against benign device profiles constructed during the initialization phase using cuckoo hash tables (Section 3.2). This process corresponds to Step 1 in Figure 2.

During profile generation, each IoT device connection is represented as a key in the hash table, while statistical traffic characteristics are stored as values. Specifically, a connection key is defined using a five-tuple: <<internal IP, external IP, destination port, protocol, direction>>. The associated values include the mean packet count and mean packet size observed for that connection. Table 1 illustrates an example IoT device profile.

To detect *connection anomalies*, unique connections observed within each traffic capture window are extracted and queried against the corresponding device profile. If a connection key does not exist in the hash table, the event is flagged as a connection anomaly, indicating previously unseen communication behavior.

If a matching key is found, the connection is further examined for *behavioral anomalies*. In this case, the observed packet statistics are compared against the stored profile values. Significant deviations in metrics such as packet count or packet size are flagged as behavioral anomalies, reflecting abnormal usage patterns over otherwise legitimate connections.

Perfect anomaly detection is not required at this stage. The primary objective is to filter benign traffic and retain potentially suspicious events for further analysis. False positives introduced during anomaly detection are subsequently refined through pattern extraction and attack-stage classification, ensuring robustness in later stages of the framework. In FDA, anomaly detection serves primarily as a filtering stage that reduces the volume of traffic forwarded to downstream pattern mining and classification modules. While occasional false positives or false negatives may occur, subsequent frequent itemset mining and association rule extraction operate on aggregated patterns rather than individual flows, which helps suppress spurious anomalies and improve robustness.

### 4.4. Pattern Extraction

Anomalies detected at the gateway are forwarded to the pattern extraction module, as indicated by Step 2 in Figure 2. The objective of this stage is to abstract heterogeneous and noisy anomaly records into compact and interpretable representations that capture recurrent attack behaviors. To achieve this, FDA employs Frequent Itemset Mining (FIM) using the Apriori algorithm.

Since FIM operates on categorical data, each anomaly is first transformed into a categorical transaction. Representative anomaly transactions are shown in Table 2. FIM is particularly suitable in this context because it identifies frequently co-occurring feature combinations without requiring labeled data, thereby reducing noise and local traffic bias while preserving attack-invariant characteristics.

***FIM Features***

- **External IP Address:** Internal IP addresses are excluded as they do not provide discriminative information. External IPs are categorized as *private* or *public*.
- **Destination Port:** Only destination ports are considered, as source ports vary dynamically. Ports not commonly associated with botnet activity are grouped under a single *any* category.
- **Protocol:** Transport-layer (TCP/UDP) and application-layer protocols (e.g., SSH, FTP, DNS, HTTP) are included when available.
- **Direction:** Traffic direction is encoded as inbound or outbound relative to the IoT device.
- **Packet Length:** Packet sizes are discretized into *small*, *medium*, and *large* categories, reflecting scanning, C&C, and volumetric attack characteristics.
- **Attack Stage:** Each transaction is labeled with one of the attack stages defined in Section 3.1. Traffic not associated with any stage is labeled as *noise*.

FIM is an unsupervised technique that extracts itemsets whose support exceeds a minimum threshold θ (0≤θ≤1). Each anomaly is represented as a *transaction*. Representative transactions are shown in Table 2. Table 3 presents selected frequent itemsets mined using a minimum support of 2/18 (Only representative patterns are shown due to space constraints). Itemsets that do not include an attack-stage attribute are excluded. The missing data and the unrecognized data are denoted by * inside the tables.

### 4.5. Association Rule Generation

The frequent itemsets obtained from the pattern extraction stage are subsequently used to derive association rules. This step establishes explicit relationships between combinations of traffic features and specific botnet attack stages. Table 4 illustrates representative association rules generated from the frequent itemsets shown in Table 3.

An association rule is expressed in the form <<X>> → <<Y>>, where X denotes the antecedent (a set of traffic features) and Y denotes the consequent. In FDA, only rules with an attack stage in the consequent and one or more traffic features in the antecedent are considered. Itemsets of size one and those without an attack-stage attribute are excluded, as they do not convey meaningful feature–stage relationships.

The strength of an association rule is quantified using its confidence, which measures the conditional probability that the consequent occurs given the antecedent. In this context, confidence reflects how strongly a particular combination of features is associated with a given attack stage. Botnet-related stages typically yield higher confidence values than benign or noise-related patterns.

To filter weak and non-informative rules, a minimum confidence threshold is applied. For example, when a threshold of 0.5 is used, association rules corresponding to noise (e.g., rules 11 and 12 in Table 4) are eliminated. The remaining rules capture strong and interpretable relationships between traffic characteristics and botnet attack stages.

In this study, a minimum support of 2/18 (≈11%) and a minimum confidence threshold of 0.5 (50%) are selected based on empirical observations from preliminary experiments to balance rule quantity, rule quality, and computational overhead. A minimum support of 2/18 suppresses extremely rare and noisy itemsets while retaining representative attack-related patterns. The minimum confidence threshold removes weak and unreliable association rules and ensures that only meaningful relationships between traffic characteristics and attack stages are retained. Such thresholding strategies are consistent with common practices in the association rule mining literature.

These high-confidence association rules serve as structured, pattern-level features for subsequent model training, enabling the classifier to learn stage-specific behaviors without relying on raw traffic data.

### 4.6. Model Training and Federated Aggregation

Although association rules can be interpreted directly, local IoT networks observe limited traffic diversity and may not encounter all attack stages or variants. To address this limitation, FDA employs federated learning to collaboratively train a classification model across distributed gateways.

Categorical features derived from association rules are transformed using one-hot encoding, resulting in a 28-dimensional input vector. A lightweight neural network is trained locally at each gateway, with six output nodes corresponding to the five attack stages and noise. Dropout layers are included to mitigate overfitting. The neural network architecture follows a sequential design with fully connected layers, LeakyReLU activations, and dropout-based regularization. The detailed configuration and parameter distribution are summarized in Table 5.

The architecture shown in Table 5 contains approximately 166 k trainable parameters, which keeps the model lightweight compared to deep neural architectures commonly used in intrusion detection. This compact design reduces memory usage and inference complexity, making deployment feasible on gateway-level devices. The baseline models are selected as representative lightweight classifiers commonly used in IoT intrusion detection studies and are suitable for comparison under gateway-level deployment assumptions, where computational resources are limited. In addition, FDA exchanges only model parameters during federated aggregation, further limiting communication overhead in distributed environments.

After local training, model parameters are transmitted to the centralized security manager via a REST interface. The security manager aggregates received parameters by averaging to construct a global model (Steps 5 and 6 in Figure 2). The aggregated model captures diverse attack characteristics observed across multiple networks while ensuring that raw traffic data remain local.

The global model is redistributed to gateways and used as the initial model for subsequent training iterations. As new IoT devices and traffic patterns emerge, gateways continue local training using newly extracted rules, periodically contributing updated parameters for aggregation. Attack-stage detection is performed locally at gateways in real time by applying the current model to patterns extracted from observed anomalies.

The choice of shallow learning models and a lightweight neural network architecture in FDA is motivated by the computational and memory constraints of IoT gateways. Deeper neural architectures and complex ensemble models typically require substantial training time, memory, and specialized hardware acceleration, which are not always available at the edge. In contrast, shallow classifiers and compact neural networks offer fast convergence and low inference latency while still providing adequate discriminative capability for pattern-level representations.

## 5. Results Evaluation and Discussion

A comprehensive set of experiments were conducted to evaluate the effectiveness of FDA. Most experiments were performed in an emulated environment using the OpenStack cloud platform to enable controlled and repeatable analysis. In addition, a practical IoT testbed experiment was carried out as a proof of concept to validate real-world applicability.

### 5.1. Experimental Setup

**Figure 3:**
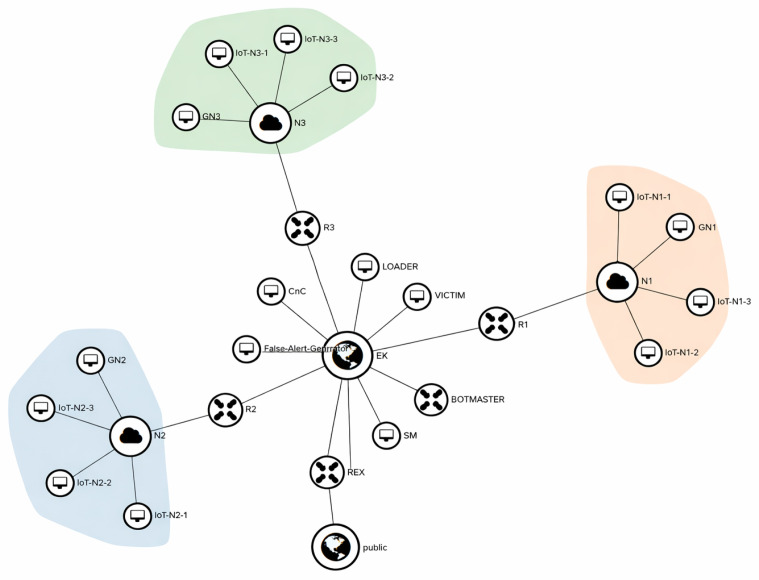
Emulated IoT network deployed in OpenStack.

An IoT network was emulated in OpenStack, as illustrated in Figure 3, and a multi-stage botnet attack resembling Mirai was implemented. The emulated environment consists of nine IoT devices, three gateways, a security manager, a botmaster, a C&C server, a malware loader, a victim machine, and a false alert generator. All components were deployed as virtual machines running Ubuntu 18.04 using custom Linux images.

All botnet attack stages described in Section 3.1 were implemented. The botmaster initiates the attack with reconnaissance, during which nmap is used for port scanning [33]. Port scanning identifies open access ports (e.g., SSH), which are treated as potential vulnerabilities. A dictionary-based brute-force login attack is then launched against these devices. Upon successful authentication, malware is retrieved from the loader via FTP and executed (The malware is registered as an init service, ensuring that the C&C channel is re-established after device reboot). This establishes persistent C&C communication with the C&C server. Finally, a SlowLoris DDoS attack is initiated via the C&C server using compromised IoT devices, targeting the victim host.

To emulate realistic operating conditions, a false alert generator produces benign background traffic throughout the experiment. This background traffic challenges the robustness of the anomaly detection and attack stage classification modules.

Four experiments were conducted using the above setup, and the corresponding results are discussed in the following subsections.

### 5.2. Experiment 01: Evaluation of Anomaly Detection

The objective of this experiment is to assess the effectiveness of the anomaly detection module. Initially, benign IoT profiles were constructed at each gateway using Cuckoo hash tables by capturing network traffic during the initial phase of normal operation. Subsequently, the botnet attack and false alert generator were activated simultaneously. While the botnet generates anomalous traffic, the false alert generator produces benign but irregular traffic patterns.

**Figure 4:**
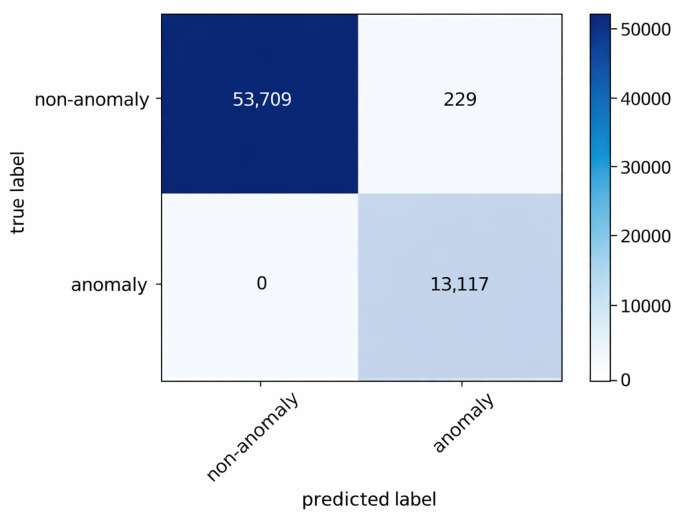
Confusion matrix of anomaly detection at gateway 1.

**Figure 5:**
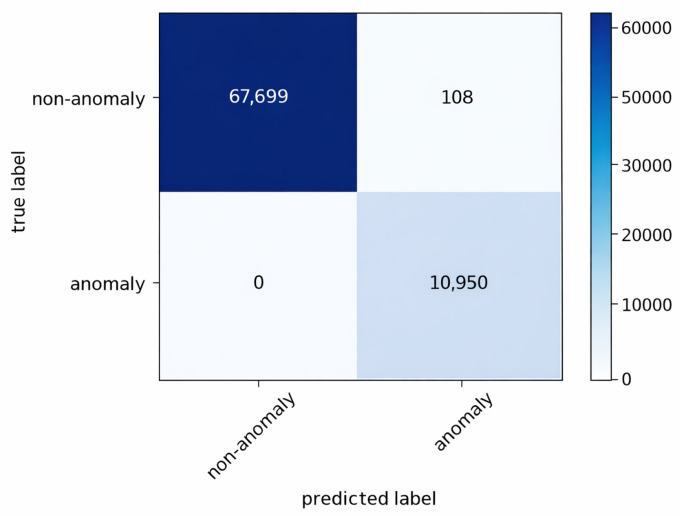
Confusion matrix of anomaly detection at gateway 2.

**Figure 6:**
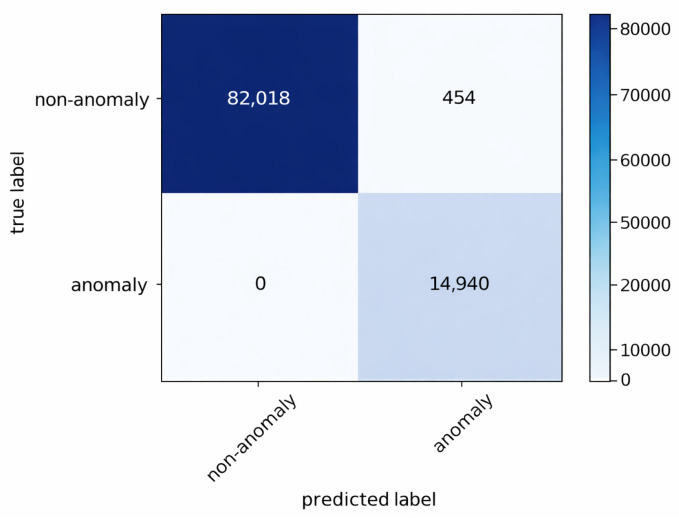
Confusion matrix of anomaly detection at gateway 3.

The anomaly detection mechanism described in Section 4.3 continuously compares observed traffic against the benign profiles. The resulting confusion matrices for the three gateways are shown in Figure 4, Figure 5 and Figure 6.

The results indicate that no anomalous connections were misclassified as benign at any gateway. Approximately 99% of all instances were correctly classified. A small number of benign flows generated by the false alert generator were incorrectly flagged as anomalies; however, these false positives are subsequently filtered during the association rule generation stage.

These results confirm that anomaly detection in FDA primarily acts as a high-recall filtering stage. Occasional false positives do not directly propagate to final attack-stage classification, as subsequent frequent itemset mining and rule extraction operate on aggregated patterns rather than individual flows.

### 5.3. Experiment 02: Evaluation of Association Rule Generation

This experiment evaluates the quality of the extracted association rules and determines suitable minimum confidence thresholds for rule selection.

Although only rules with attack stages in the consequent are considered, the total number of generated rules can still be large, including uninformative rules. To assess rule usefulness, the lift metric is employed, defined as

(1) $$\mathrm{Lift}\left(X\to Y\right)=\frac{\sup(X,Y)}{\sup(X)\sup(Y)},$$

where *X* and *Y* denote itemsets and sup(·) represents support.

A rule is considered *interesting* if its lift is greater than or equal to one. Rules satisfying this condition are referred to as positive rules; otherwise, they are classified as negative rules.

**Figure 7:**
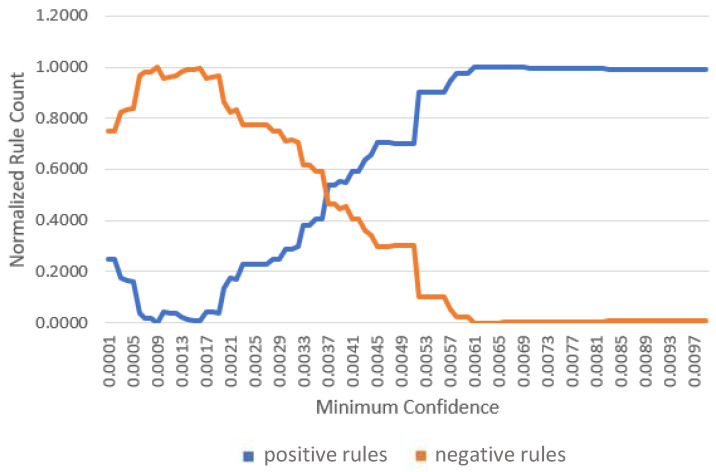
Positive and negative rule counts versus minimum confidence at gateway 1.

**Figure 8:**
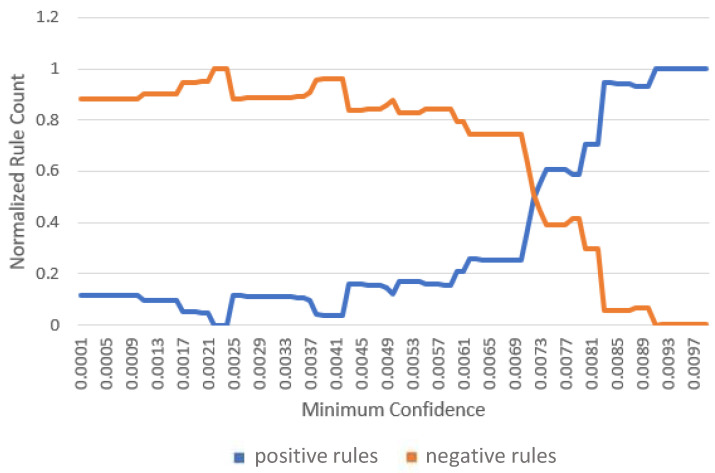
Positive and negative rule counts versus minimum confidence at gateway 2.

**Figure 9:**
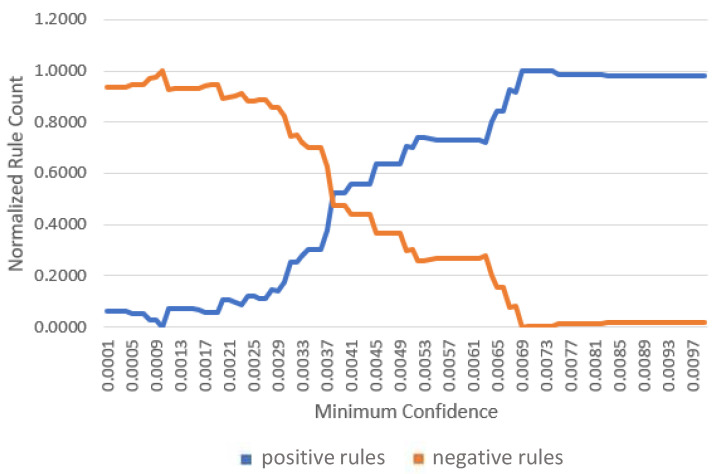
Positive and negative rule counts versus minimum confidence at gateway 3.

For each gateway, association rules were evaluated across 200 different minimum confidence values. The proportions of positive and negative rules were computed and plotted against the confidence threshold. The results are shown in Figure 7, Figure 8 and Figure 9.

As expected, increasing the minimum confidence threshold results in a higher proportion of positive rules and a corresponding reduction in negative rules. Both trends saturate beyond specific confidence levels, identified as 0.0061, 0.0091, and 0.007 for gateways one, two, and three, respectively. Although higher confidence thresholds yield a greater fraction of interesting rules, the absolute number of retained rules decreases. Therefore, the identified saturation points are selected as the minimum confidence thresholds for subsequent experiments.

### 5.4. Experiment 03: Evaluation of Decentralized and Centralized Approaches

This experiment compares the performance of centralized and decentralized (federated) training strategies. Five test cases were considered. For each test case, the local dataset at each gateway was randomly partitioned, and each partition was split into training and testing subsets using a 70:30 ratio.

For the centralized approach, training subsets from all gateways were aggregated to form a single global training dataset, while the corresponding testing subsets were combined to form a centralized test set. A centralized neural network model with the same architecture shown in Figure 5 was trained on this aggregated dataset. This approach requires transferring local data to a central entity and is therefore privacy intrusive.

In contrast, the proposed FDA framework adopts a decentralized strategy, where models are trained locally at gateways using their respective datasets. Only the trained model weight parameters are shared with the security manager, which aggregates them to produce a global model. No raw traffic data leave the gateways.

**Figure 10:**
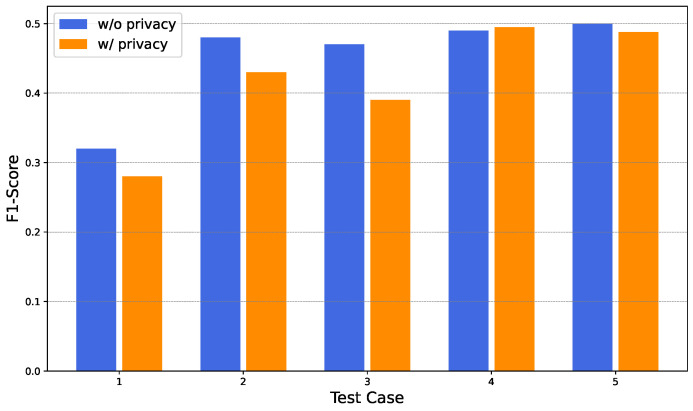
Performance comparison between centralized and decentralized approaches.

The F1-score (The F1-score is the harmonic mean of precision and recall) was used as the evaluation metric. The results are presented in Figure 10.

The centralized approach is labeled as *without privacy* in Figure 10, as it requires sharing user data with a third-party entity. The decentralized approach is labeled as *with privacy*, since user data remain at the gateways.

The centralized model achieves slightly higher F1-scores in four out of five test cases, while the decentralized model marginally outperforms the centralized model in one case. Overall, the average performance difference between the two approaches is approximately 0.025, demonstrating that FDA achieves competitive detection performance while preserving data privacy.

### 5.5. Experiment 04: Comparison with Existing Methods

In this experiment, the performance of FDA is compared with classical supervised learning algorithms, namely Support Vector Machine (SVM), K-Nearest Neighbors (KNN), and Stochastic Gradient Descent (SGD). These baseline methods were trained in a centralized manner using the aggregated dataset, whereas FDA operates in a decentralized fashion.

All baseline classifiers (SVM, KNN, and SGD) are trained using the same feature representation, dataset partitions, and evaluation protocol as FDA to ensure fair comparison. Standard parameter settings are adopted for these models to reflect lightweight deployment scenarios and to avoid excessive model complexity. In all experiments, benign background traffic generated by the false alert generator is continuously present, introducing realistic noise. The impact of this noise is partially reflected in the anomaly detection confusion matrices and propagates to the subsequent stage-wise classification results.

**Figure 11:**
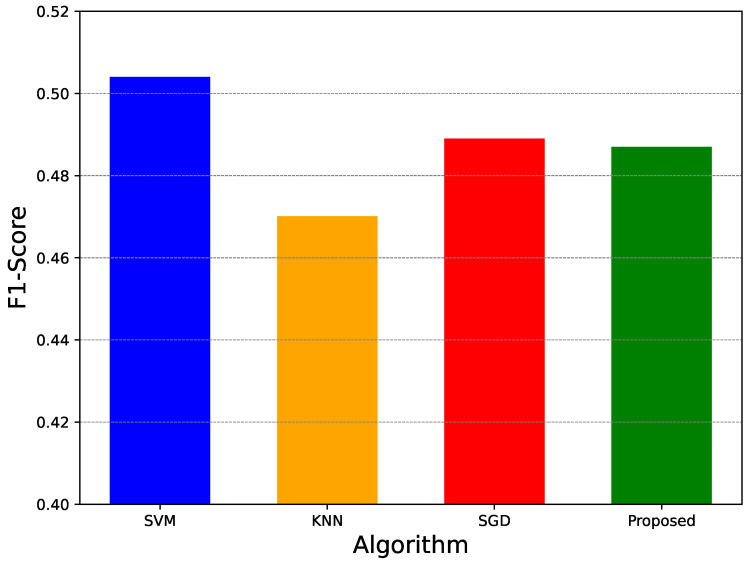
F1-score comparison with existing classification.

Consistent with the previous experiments, the F1-score was used as the evaluation metric. The comparative results are shown in Figure 11.

Among the evaluated methods, SVM achieves the highest F1-score, while KNN exhibits the lowest performance. FDA and SGD achieve F1-scores in the range of 48–49%. Notably, the difference between the best-performing centralized model (SVM) and FDA is less than 2%, indicating that FDA provides competitive classification accuracy despite operating under strict privacy constraints.

Baseline models such as SVM and SGD were selected as reference points because they are widely used in intrusion detection systems and represent common trade-offs between accuracy and computational cost. By demonstrating performance comparable to these baselines while operating under federated and decentralized constraints, FDA highlights that the adopted lightweight design inherently contributes to a low-cost and deployable solution.

Baseline models such as SVM, KNN, and SGD are included in this study because classical machine-learning classifiers are widely adopted as benchmark models in IoT intrusion detection research due to their strong performance and low computational complexity [34,35]. Prior studies show that traditional classifiers often provide competitive results in IoT network traffic classification and therefore remain important reference points when evaluating newly proposed approaches.

Accordingly, the comparison presented in Figure 11 and Table 6 provides quantitative positioning of FDA with respect to representative learning approaches commonly used in IoT IDS studies. The results show that FDA achieves comparable F1-scores to centralized baseline models while operating under decentralized and privacy-preserving constraints. This highlights the trade-off between centralized learning, which relies on direct access to aggregated data, and the proposed decentralized framework, which maintains data locality while preserving competitive detection performance.

Unlike the centralized baselines, FDA does not require direct access to user data for training, as it relies solely on locally trained model parameters. This highlights the effectiveness of FDA in balancing detection performance and data privacy.

It is important to note that multi-stage botnet attack classification is inherently more challenging than binary intrusion detection, as multiple attack stages may exhibit overlapping traffic characteristics and class imbalance. In operational environments, the primary objective of FDA is to provide early-stage awareness and visibility into attack progression rather than perfect per-flow classification. From this perspective, F1-scores in the range of 48–50% are practically useful, as they enable security operators to correlate evolving attack stages, trigger further inspection, and initiate mitigation actions. These results therefore reflect a deliberate trade-off between fine-grained classification accuracy, privacy preservation, and scalable deployment.

### 5.6. Proof of Concept Using a Real IoT Testbed

A real IoT testbed was constructed to validate the practicality of FDA under realistic conditions. A Mirai-like botnet attack was implemented as a proof-of-concept experiment. The objective of this experiment is to demonstrate the feasibility of deploying FDA in a physical environment and to verify that the proposed pipeline can operate in real time using flow-level traffic features.

**Figure 12:**
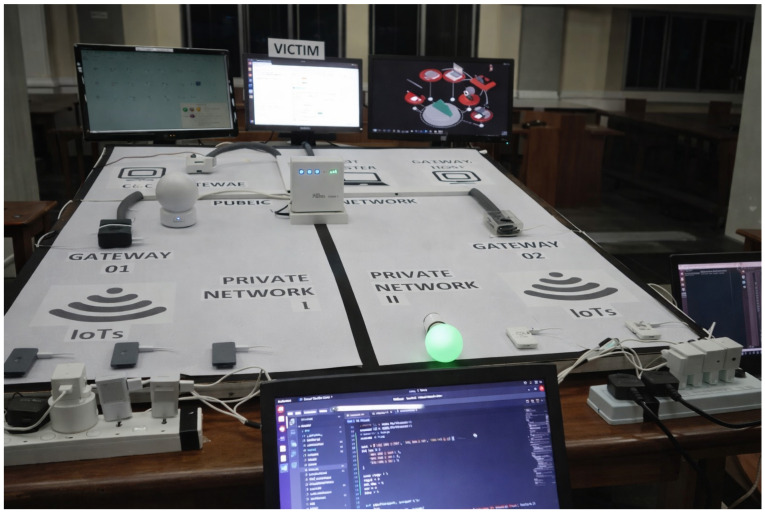
Physical IoT testbed used for evaluation.

The testbed consists of eight IoT devices, two gateways, a botmaster, a C&C server, a malware loader, a victim machine, a router, a switch, and a security manager, as illustrated in Figure 12. The botmaster, victim, and security manager were implemented using personal computers, while the gateways, C&C server, and loader were deployed on Raspberry Pi boards. In addition to real IoT devices such as smart cameras, smart bulbs, and smart plugs, five Raspberry Pi boards were configured to emulate IoT devices. All Raspberry Pi devices and gateways run lightweight Linux-based operating systems and host common network services such as SSH and HTTP to emulate realistic IoT communication behavior.

The router acted as the network head node and assigned IP addresses to all devices. To ensure safety, the testbed was isolated from external networks by disabling internet connectivity.

**Figure 13:**
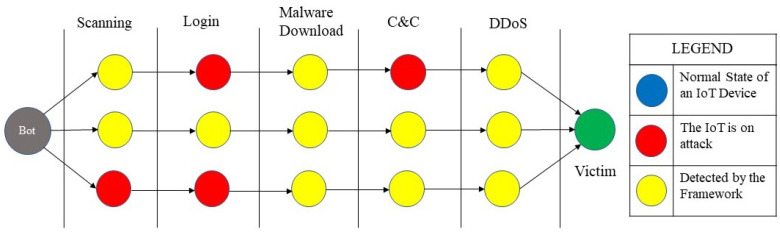
GUI illustrating detected botnet attack stages.

All attack stages defined in Section 3.1 were executed in the testbed. The same FDA pipeline described in Section 4 was applied, and detected attack stages were visualized using a graphical user interface, as shown in Figure 13.

During each experiment, the testbed was first operated under benign conditions to establish baseline device profiles. Subsequently, attack stages were launched sequentially, including scanning, brute-force login, malware download, C&C communication, and DDoS traffic generation. Traffic was captured at the gateways, processed by the FDA pipeline, and the detected stages were compared with ground-truth attack logs.

Based on visual analysis of the real testbed demonstration in Figure 13, FDA detects the majority of executed botnet attack-stage instances across all phases. Approximate stage-detection rates observed are about 80% for reconnaissance, 75% for login, 85% for malware infection, 80% for C&C communication, and 90% for DDoS. In addition, all five attack stages are detected at both gateways, and the final DDoS stage targeting the victim is correctly identified, demonstrating reliable end-to-end attack-stage visibility in the physical deployment.

Although the real testbed is not intended for large-scale benchmarking, these results, together with the quantitative anomaly detection performance reported in Section 5.2 (F1-scores above 0.99), provide evidence that FDA can operate reliably in real environments. Furthermore, because FDA performs local training at gateways and exchanges only model parameters through federated aggregation, the framework can scale to additional gateways and IoT networks without requiring centralized traffic collection.

Overall, the results in Figure 9 and Figure 10 demonstrate that FDA achieves detection performance that is closely comparable to centralized machine-learning baselines, despite operating under decentralized and privacy-preserving constraints. While centralized SVM achieves the highest F1-score, the performance gap between SVM and FDA is less than 2%, indicating that pattern-level abstraction combined with federated learning preserves most of the discriminative power of centralized training.

FDA is implemented as a modular pipeline consisting of anomaly detection, discretization, frequent itemset mining, association rule extraction, and local model training. Although this introduces multiple tunable parameters (e.g., anomaly thresholds, discretization ranges, and support/confidence values), these parameters are selected through empirical evaluation and remain fixed during normal operation. To maintain detection reliability under evolving traffic conditions, local models are periodically retrained using newly observed patterns, and federated aggregation is performed at regular intervals to refresh the global model. While pipeline-based approaches inherently introduce additional maintenance effort, FDA employs lightweight components and simple update mechanisms that help keep the operational overhead manageable.

### 5.7. Overall Results Discussion

The competitive performance of FDA can be attributed to two main factors. First, the frequent itemset mining and association rule generation stages transform heterogeneous and noisy anomalies into compact, high-confidence patterns, thereby reducing the impact of local traffic bias and class imbalance. Second, federated aggregation enables gateways to collaboratively learn stage-specific behaviors observed across multiple networks, which improves generalization without exposing raw traffic data.

Although the achieved F1-scores for multi-stage classification (approximately 48–49%) reflect the intrinsic difficulty of fine-grained attack-stage discrimination in IoT environments, they remain practical for early-warning and situational-awareness purposes.

Overall, these findings confirm that FDA offers a favorable trade-off between detection accuracy, scalability, and privacy, providing performance comparable to centralized estimators while eliminating the need for sharing sensitive network data.

It is also important to note that FDA relies exclusively on flow-level features and does not inspect packet payloads. While this design choice is motivated by privacy preservation and deployment practicality, it may limit the ability to detect certain content-dependent or highly obfuscated attacks that require deep packet inspection. Nevertheless, the results demonstrate that pattern-level representations derived from flow features capture sufficient behavioral information to achieve competitive detection performance, highlighting a practical trade-off between detection granularity and privacy.

## 6. Conclusions

This paper introduced FDA for stage-aware botnet attack detection in IoT networks. FDA was designed to address key limitations of existing centralized intrusion detection approaches, particularly privacy risks, traffic heterogeneity, and limited visibility across distributed IoT deployments.

By combining network-level anomaly detection with Frequent Itemset Mining and association rule analysis, FDA transforms raw anomalous traffic into compact and interpretable pattern representations that are suitable for collaborative learning. Lightweight neural network models trained locally at gateways enable attack-stage classification without direct exposure of user traffic, while federated aggregation allows the global model to learn from diverse attack manifestations observed across multiple IoT networks.

Experimental evaluation using an OpenStack-based emulation environment demonstrated that the anomaly detection component of FDA is highly reliable, achieving F1-scores above 0.99 across all gateways. For multi-stage botnet attack classification, FDA achieved F1-scores in the range of 0.48–0.49, which are comparable to centralized machine-learning baselines such as SVM and SGD, despite operating under decentralized and privacy-preserving constraints. The observed performance gap between federated and centralized training remained small (approximately 0.025 in terms of F1-score), indicating that FDA preserves competitive detection capability while avoiding direct data aggregation.

A real IoT testbed experiment further validated the practical applicability of the proposed framework, where FDA successfully detected and distinguished multiple botnet attack stages under realistic traffic conditions. These results suggest that, although fine-grained stage-level classification remains challenging due to overlapping traffic characteristics, class imbalance, and the evolving nature of IoT botnet behaviors, FDA provides a viable and scalable solution for distributed botnet attack analysis in privacy-sensitive IoT environments.

From a deployment perspective, FDA can be integrated into IDS, SOC, and XDR platforms by operating gateway-level FDA components as distributed sensors that forward stage-aware alerts and federated model updates to a central security analytics engine. Such integration would enable correlation of FDA outputs with logs from firewalls, SIEM systems, and endpoint security tools, providing unified situational awareness and supporting automated response workflows.

Future work will focus on improving stage discrimination for subtle and low-volume attack behaviors, incorporating adaptive or retrieval-based mechanisms to better handle previously unseen attack patterns, and evaluating the framework at larger scales with more diverse IoT deployments. Additionally, optimizing model efficiency and communication overhead will be explored to further enhance the practicality of FDA in resource-constrained edge environments.

## Figures and Tables

**Table 1:** An emulated profile of an IoT device.

Key					Value	
**int_IP**	**ext_IP**	**dst_Port**	**Protocol**	**Dir**	**Count**	**Size**
172.24.4.159	10.1.0.104	8080	TCP	OUT	11	76
172.24.4.159	10.1.0.104	44,634	TCP	IN	1	56
172.24.4.159	10.1.0.104	44,635	TCP	IN	1	56
172.24.4.159	10.1.0.104	44,636	TCP	IN	1	56

**Table 2:** Sample input to FIM.

#	External IP	Destination Port	Protocol	Dir	Packet Length	Attack Stage
1	Private	22	SSH	IN	Small	Scan
2	Private	22	SSH	IN	Small	Scan
3	Private	23	Telnet	IN	Small	Scan
4	Private	22	SSH	IN	Medium	Login
5	Private	22	SSH	IN	Small	Login
6	Public	21	FTP	OUT	Large	Malware
7	Public	21	FTP	OUT	Large	Malware
8	Private	21	TCP	IN	Small	Malware
9	Private	7000	TCP	OUT	Medium	C&C
10	Private	7000	TCP	OUT	Medium	C&C
11	Private	7000	TCP	IN	Small	C&C
12	Public	80	HTTP	OUT	Large	DDoS
13	Public	80	HTTP	OUT	Large	DDoS
14	Public	53	DNS	OUT	Small	DDoS
15	Public	53	DNS	OUT	Small	DDoS
16	Private	Any	TCP	OUT	Medium	Noise
17	Private	443	TCP	IN	Small	Noise
18	Private	28	UDP	IN	Large	Noise

**Table 3:** Representative frequent itemsets extracted from Table 2.

#	Ext. IP	Dst. Port	Protocol	Dir	Pkt. Length	Stage	Support
1	Private	*	SSH	IN	Small	Scan	2/18
2	Private	22	SSH	IN	Small	Scan	2/18
3	Private	22	SSH	IN	*	Login	2/18
4	Public	21	FTP	OUT	Large	Malware	2/18
5	*	21	*	*	*	Malware	3/18
6	Private	7000	TCP	OUT	Medium	C&C	2/18
7	Private	7000	TCP	*	*	C&C	3/18
8	Public	80	HTTP	OUT	Large	DDoS	2/18
9	Public	53	DNS	OUT	Small	DDoS	2/18
10	Public	*	*	OUT	*	DDoS	4/18

**Table 4:** Representative association rules generated from the frequent patterns in Table 3.

#	Antecedent	Consequent	Confidence
1	<<Private, *, SSH, IN, Small>>	Scan	0.66
2	<<Private, 22, SSH, IN, Small>>	Scan	0.66
3	<<Private, 22, SSH, IN, *>>	Login	0.50
4	<<Public, 21, FTP, OUT, Large>>	Malware	1.00
5	<<*, 21, *, *, *>>	Malware	1.00
6	<<Private, 7000, TCP, OUT, Medium>>	C&C	1.00
7	<<Private, 7000, TCP, *, *>>	C&C	1.00
8	<<Public, 80, HTTP, OUT, Large>>	DDoS	1.00
9	<<Public, 53, DNS, OUT, Small>>	DDoS	1.00
10	<<Public, *, *, OUT, *>>	DDoS	0.66
11	<<Private, *, TCP, *, *>>	Noise	0.33
12	<<Private, *, *, IN, *>>	Noise	0.22

**Table 5:** Detailed architecture of the neural network used for local classification.

Layer (Type)	Output Shape	Parameters
Dense	(None, 20)	580
Dense	(None, 200)	4200
LeakyReLU	(None, 200)	0
Dropout	(None, 200)	0
Flatten	(None, 200)	0
Dense	(None, 400)	80,400
LeakyReLU	(None, 400)	0
Dropout	(None, 400)	0
Flatten	(None, 400)	0
Dense	(None, 200)	80,200
LeakyReLU	(None, 200)	0
Dropout	(None, 200)	0
Dense	(None, 6)	1206
Total Parameters		166,586
Trainable Parameters		166,586
Non-trainable Parameters		0

**Table 6:** Quantitative positioning of FDA against representative approaches.

Method	Training Strategy	F1-Score
SVM (centralized)	Centralized	0.50
SGD (centralized)	Centralized	0.48
KNN (centralized)	Centralized	0.42
FDA (proposed)	Federated	0.48–0.49

## Data Availability

The data presented in this study are available on request from the corresponding author due to privacy and security restrictions related to IoT network traffic.

## References

[1] (2021). Fortune Business Insights. Internet of Things (IoT) Market. https://www.fortunebusinessinsights.com/industry-reports/internet-of-things-iot-market-100307.

[2] Kumar D., Shen K., Case B., Garg D., Alperovich G., Kuznetsov D., Gupta R., Durumeric Z. (2019). All Things Considered: An Analysis of IoT Devices on Home Networks. Proceedings of the 28th USENIX Security Symposium.

[3] Antonakakis M., April T., Bailey M., Bernhard M., Bursztein E., Cochran J., Durumeric Z., Halderman J.A., Invernizzi L., Kallitsis M. (2017). Understanding the Mirai Botnet. Proceedings of the 26th USENIX Security Symposium, Vancouver, BC, Canada, 16–18 August 2017.

[4] Divakaran D.M., Singh R.P., Sudheera K.L.K., Gurusamy M., Sachidananda V. (2020). ADROIT: Detecting Spatio-Temporal Correlated Attack-Stages in IoT Networks. Proceedings of the NDSS-DISS.

[5] Apthorpe N., Reisman D., Feamster N. (2017). A Smart Home Is No Castle: Privacy Vulnerabilities of Encrypted IoT Traffic. arXiv.

[6] Acar A., Fereidooni H., Abera T., Sikder A.K., Miettinen M., Aksu H., Conti M., Sadeghi A.-R., Uluagac S. (2020). Peek-a-Boo: I See Your Smart Home Activities, Even Encrypted!. Proceedings of the ACM WiSec.

[7] Anthi E., Williams L., Słowińska M., Theodorakopoulos G., Burnap P. (2019). A Supervised Intrusion Detection System for Smart Home IoT Devices. IEEE Internet Things J..

[8] Nømm S., Bahşi H. (2018). Unsupervised Anomaly Based Botnet Detection in IoT Networks. Proceedings of the IEEE ICMLA.

[9] Samy A., Yu H., Zhang H. (2020). Fog-Based Attack Detection Framework for IoT Using Deep Learning. IEEE Access.

[10] Alani M.M. (2021). Detection of Reconnaissance Attacks on IoT Devices Using Deep Neural Networks. Advances in Nature-Inspired Cyber Security and Resilience.

[11] Siadati H., Memon N. Detecting Structurally Anomalous Logins Within Enterprise Networks. Proceedings of the ACM CCS.

[12] Hofstede R., Hendriks L., Sperotto A., Pras A. (2014). SSH Compromise Detection Using NetFlow/IPFIX. SIGCOMM Comput. Commun. Rev..

[13] Ghafir I., Prenosil V., Hammoudeh M., Baker T., Jabbar S., Khalid S., Jaf S. (2018). BotDet: A System for Real-Time Botnet Command and Control Traffic Detection. IEEE Access.

[14] Doshi R., Apthorpe N., Feamster N. (2018). Machine Learning DDoS Detection for Consumer Internet of Things Devices. Proceedings of the IEEE Security and Privacy Workshops (SPW).

[15] Hamza A., Gharakheili H.H., Benson T.A., Sivaraman V. (2019). Detecting Volumetric Attacks on IoT Devices via SDN-Based Monitoring of MUD Activity. Proceedings of the ACM SOSR.

[16] Valdes A., Skinner K. (2001). Probabilistic Alert Correlation. Proceedings of the RAID.

[17] Brahmi H., Ben Yahia S. (2013). Discovering Multi-Stage Attacks Using Closed Multi-Dimensional Sequential Pattern Mining. Proceedings of the DEXA.

[18] Ourston D., Matzner S., Stump W., Hopkins B. (2003). Applications of Hidden Markov Models to Detecting Multi-Stage Network Attacks. Proceedings of the 36th Annual Hawaii International Conference on System Sciences.

[19] Shin J., Choi S.-H., Liu P., Choi Y.-H. (2019). Unsupervised Multi-Stage Attack Detection Framework. Future Gener. Comput. Syst..

[20] Sudheera K.L.K., Divakaran D.M., Singh R.P., Gurusamy M. (2021). ADEPT: Detection and Identification of Correlated Attack Stages in IoT Networks. IEEE Internet Things J..

[21] Trimananda R., Varmarken J., Markopoulou A., Demsky B. (2020). Packet-Level Signatures for Smart Home Devices. Proceedings of the NDSS.

[22] Konečný J., McMahan H.B., Ramage D., Richtárik P. (2016). Federated Optimization: Distributed Machine Learning for On-Device Intelligence. arXiv.

[23] Boddy S., Shattuck J., Walkowski D., Warburton D. The Hunt for IoT: Multi-Purpose Attack Thingbots Threaten Internet Stability and Human Life. F5 Labs Technical Report, 2018. https://www.f5.com/labs/articles/threat-intelligence/the-hunt-for-iot--multi-purpose-attack-thingbots-threaten-intern.

[24] Kolias C., Kambourakis G., Stavrou A., Voas J. (2017). DDoS in the IoT: Mirai and Other Botnets. Computer.

[25] Bailey M., Cooke E., Jahanian F., Xu Y., Karir M. (2009). A Survey of Botnet Technology and Defenses. Proceedings of the Cybersecurity Applications & Technology Conference for Homeland Security.

[26] TutorialsPoint Ethical Hacking—DDoS Attacks. https://www.tutorialspoint.com/ethical_hacking/ethical_hacking_ddos_attacks.htm.

[27] Ahmed T., Oreshkin B., Coates M. (2007). Machine Learning Approaches to Network Anomaly Detection. Proceedings of the USENIX Workshop on ML.

[28] Raschka S. (2018). MLxtend: Machine Learning and Data Science Utilities. J. Open Source Softw..

[29] Agrawal R., Srikant R. Fast Algorithms for Mining Association Rules. Proceedings of the 20th International Conference on Very Large Data Bases (VLDB).

[30] Klosowski T. The State of Consumer Data Privacy Laws in the US (And Why It Matters). *The New York Times Wirecutter*, 2021. https://www.nytimes.com/wirecutter/blog/state-of-privacy-laws-in-us/.

[31] ShieldIOT ShieldIOT: Gateway Security. https://stage.shieldiot.io/device-level-security/.

[32] Bhatt S., Manadhata P.K., Zomlot L. (2014). The Operational Role of Security Information and Event Management Systems. IEEE Secur. Priv..

[33] Port Scanners Port Scanners Infosec Resources. https://www.infosecinstitute.com/resources/penetration-testing/port-scanners/.

[34] Karunamurthy A., Vijayan K., Kshirsagar P.R. (2025). An Optimal Federated Learning-Based Intrusion Detection for IoT Environment. Sci. Rep..

[35] Churcher A., Ullah R., Ahmad J., Rehman S.u., Masood F., Gogate M., Alqahtani F., Nour B., Buchanan W.J. (2021). An Experimental Analysis of Attack Classification Using Machine Learning in IoT Networks. Sensors.

